# The General and Special Education of Dentists

**Published:** 1881-12

**Authors:** 


### EDITORIAL DEPARTMENT.

The General and Special Education of Dentists.—In our leading editorial in the November number of the *Independent Practitioner* we stated the views held by us on the present standing of the question of medical education. Regarding dentistry merely as a branch or specialty of general medicine, we hold that in certain of the general branches the education of medical and dental students should be similar. At a certain point of the curriculum the training of the two classes of students should diverge and become special. When this divergence of studies should take place we shall try to indicate in this paper.

As regards the preliminary examination the requirements should be the same for the two classes of students. The dentist must be able to understand the laws of physics and chemistry as well as the medical student, and he should be on the same plane with him as regards general culture.

A knowledge of chemistry, anatomy and physiology is as important to the dentist as to the physician. The first and third should be taught throughout in a similar manner to both medical and dental students, the lectures and laboratory work being the same for both. In anatomy the lectures should also be the same, while the practical work in the dissecting room should here be specialized, the dentist devoting most of his time in the dissecting room to the study of the parts with which he will in his future practice be more immediately concerned.

General pathology should not be distinctively medical or dental, for the processes of inflammation hypertrophy, atrophy, degeneration and new-formation do not essentially differ, whether they occur in the mouth or in the genital organs. Special pathology should, however, be specialized as much as possible, for the dental student would profit little by attending the lectures on ophthalmology, gynæcology, or any of the other specialties except dentistry. He must learn all about the diseases affecting the mouth, teeth and jaws, and be able to treat these diseases and obviate their unpleasant results. It matters little whether he knows anything of internal urethrotomy or hysterotrachelorraphy, or whether he is entirely ignorant of these advances in modern special surgery.

Then, instead of the medical student's special training in surgical operations, or obstetric work, the dental student must, by demonstration and practice become practically acquainted with the operative and artificial branches of dentistry. His instruction in these should begin from the time he enters the dental infirmary, for they are to him what the hospital work and operations are to his brother of the medical school.

The question now remains how best to secure the most thorough acquirement of this knowledge. It is our belief that a similar plan to that urged in relation to the education of medical students should be pursued in dental schools. The general branches, anatomy, physiology, chemistry, materia medica, with practical exercises in oral surgery, laboratory and dissecting room should be taught the first year, and the amount of knowledge acquired tested by an examination at the end of the term. The second year's course should be principally occupied with special pathology and operations. Both the theory and practice of operative and artificial dentistry should be taught throughout both years, and as much time as possible be given to general practice.

Habits of cleanliness—of body, clothing and surroundings—should, of course, have been formed before the student enters the college, but those who, like the writer, have had experience as an instructor in a dental school, know how often this important part of the preliminary education of a dentist is neglected. It is therefore incumbent upon the teacher to constantly enforce upon the student, both by precept and example, the necessity of being scrupulously clean in all things. He should be impressed with the fact that thorough cleanliness is essential to success in dentistry above all professions.

If the methods of medical and dental teaching, which we have attempted to outline were adopted, it is believed that it would considerably raise the standard of medical education in this country. At the same time it would involve no radical or injurious change to the colleges; on the other hand it would necessitate harder study on the part of the students, and better teaching on the part of the teachers.

